# Optimized flow cytometric protocol for the detection of functional subsets of low frequency antigen-specific CD4^+^ and CD8^+^ T cells

**DOI:** 10.1016/j.mex.2020.101005

**Published:** 2020-07-22

**Authors:** Marie Mura, Sidhartha Chaudhury, Fouzia Farooq, Elizabeth H. Duncan, Kevin Beck, Elke S. Bergmann-Leitner

**Affiliations:** a Malaria Biologics Branch, Walter Reed Army Institute of Research, Silver Spring, MD 20910, United States; b Anti-Infectious Biotherapies and Immunity, Institut de Recherche Biomédicale des Armées, BP73, Brétigny-sur-Orge Cedex, France; c Data Analytics Core, Walter Reed Army Institute of Research, Silver Spring, MD, United States; d Lentigen Technology, Inc. Gaithersburg, MD 20878, United States

**Keywords:** Flow cytometry, Intracellular staining, Cell enrichment, Antigen-specific cell detection, Rare cell detection, Cytokine response

## Abstract

Detection of low-frequency cells using flow cytometry is challenging, as the sensitivity of the analysis is dependent on the signal-to-noise ratio, and a cell frequency of 1 in 10,000 cells is accepted as the lower limit of detection for standard flow cytometry. A solution to this problem is to pre-enrich rare cell populations using magnetic-bead conjugated antibodies targeting lineage or activation markers. For measuring vaccine or pathogen induced immune responses, this method drastically increases the signal-to-noise ratio by enriching only activated (i.e., antigen-specific) cells and excluding all other peripheral blood leukocytes from the subsequent analysis. To date, magnetic enrichment of antigen-specific cells has only been described for CD4^+^ T cells processed for surface staining. The current study significantly expands the methodology to allow detection of antigen-specific CD8^+^ T cells and analysis of cells that had been processed for intracellular staining.

• The protocol described here allows magnetic enrichment of PBMCs after fixation and intracellular staining steps without increasing the non-specific background.
• The protocol is adapted to automated enrichment-mode on flow cytometers.
• The procedure boosts the sensitivity of the flow cytometry analysis by significantly increasing the sample size of functional antigen-specific cells without skewing the composition of the functional cells pool.

## Graphical abstract

**Figure:**
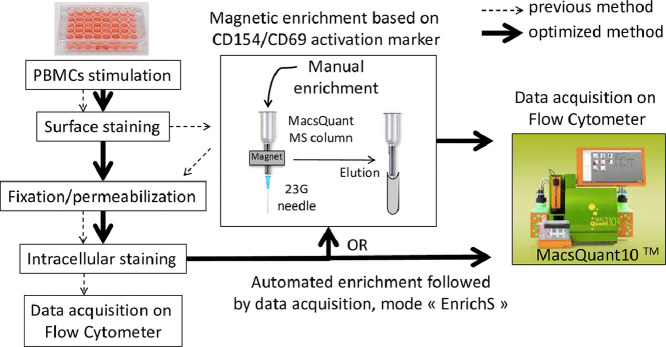

Specifications Table

**Table:** 

Subject area	Immunology and microbiology
More specific subject area:	Single cell analysis by flow cytometry
Method name:	Magnetic enrichment and flow cytometry
Name and reference of original method:	Miltenyi S, Muller W, Weichel W, Radbruch A. High gradient magnetic cell separation with MACS. Cytometry. 1990;11(2):231–8.
Resource availability	All reagents and equipment are listed with the name of the suppliers.

## Method details

### Reagents

1. Cryopreserved PBMCs
2. Complete medium (cRPMI)

RPMI 1640 (Life Technologies, Waltham, MA)

Penicillin-Streptomycin (1:100) (Quality Biological, Gaithersburg, MD)

Non-Essential Amino Acids (1:100) (Quality Biological, Gaithersburg, MD)

L-glutamine (1:100) (Quality Biological, Gaithersburg, MD)

Sodium Pyruvate (1:100) (Quality Biological, Gaithersburg, MD)

2-Mercaptoethanol (1:1000) (Life Technologies, Waltham, MA)

Commercial human AB serum (10%) (Gemini Bio-Products, West Sacramento, CA)

3. Flow Buffer

500 mL 1X DPBS (Life Technologies, Waltham, MA)

0.01% Sodium Azide

1% Human AB Serum (Gemini Bio-Products, West Sacramento, CA)

4. Manual separation buffer (make fresh, degas either using vacuum or water bath)

1X DPBS (Life Technologies, Waltham, MA)

0.5% BSA (Thermo Fisher scientific, Waltham, MA)

2 mM EDTA (Quality Biological, Gaithersburg, MD)

5. 1X DPBS (Life Technologies, Waltham, MA)
6. Fixation/ Permeabilization kit (BD Pharmingen, San Jose, CA) with Brefeldin A
7. Antibodies:

anti-human CD40 pure (clone: HB14, Miltenyi Biotec) anti-human CD154-biotin (clone 5C8, Miltenyi Biotec) anti-human CD69-biotin (clone REA824, Miltenyi Biotec) anti-biotin MicroBeads, ultrapure (Miltenyi Biotec) anti-human CD3 Vioblue (clone BW264/56, Miltenyi Biotec) anti-human CD4 PerCP-Vio700 (clone M-T466, Miltenyi Biotec) anti-human CD8 APC-Vio770 (clone BW135/80, Miltenyi Biotec) anti-biotin PE (clone Bio3–18E7, Miltenyi Biotec)

Zombie Aqua Fixable dye for viability (BioLegend, San Diego, CA) anti-human IFN-γ FITC (clone 54–15, Miltenyi Biotec)

8. Antigens:

Tetanus toxoid (1 µg/ml)

CEF-Class I peptide pool plus (2 µg/ml) (Cellular Technology Limited, Shaker Heights, OH)

CEF-Class II peptide pool plus (4 µg/ml) (PANATecs GmbH, Tübingen, Germany)

Staphylococcus enterotoxin B (0.5 µg/ml)

Cytostim™ reagent (20 µl/ml) (Miltenyi Biotec, San Diego, CA)

9. MS or LS separation columns (Miltenyi Biotec, San Diego, CA)
10. 23 g syringe needles
11. MACSQuant Calibration beads (Miltenyi Biotec, San Diego, CA)

### Equipment

1. OctoMACS magnet (Miltenyi Biotec, San Diego, CA) for manual enrichment
2. MACSQuant Analyzer 10 (Miltenyi Biotec, San Diego, CA) for automated enrichment mode “EnrichS” and data acquisition

## Procedure

### Cell stimulation

1. Peripheral blood mononuclear cells were obtained from de-identified donors of a blood collection protocol (WRAIR#2567) conducted at the Clinical Trials Center at WRAIR using standard method of density gradient separation with Lymphocyte Separation Medium LSM^Ⓡ^ (MP Biomedicals, LLC, OH).
2. Cells are cultured for 18 h (37 °C, 5% CO_2_) in cRPMI at a concentration of 5 × 10^6^ cells/mL with the listed antigens or in medium alone (negative control).
3. Anti-CD28 and CD49d costimulatory monoclonal antibodies can be used to enhance the T-cell stimulation if desired at a concentration of 1 µg/ml.
4. CD40 pure antibody (clone: HB14, Miltenyi Biotec) is added to the culture at a 1:100 dilution according to the manufacturer's recommendation. Brefeldin A is added after 2 h of stimulation in order to inhibit the release of cytokines.

### Cell processing (staining and fixation/permeabilization)

5. Put the cells on ice 15 min
6. Centrifuge cells (350 g, 6 min, 4 °C), wash cells with cold Flow Buffer and centrifuge again (350 g, 6 min, 4 °C)
7. Resuspend the pellet in CD154 or CD69-biotin antibody in Flow Buffer (15 min, +4 °C) depending on the goal of the study (CD154 for enrichment of activated CD4^+^T cells, CD69 for enrichment of both CD4^+^ and CD8^+^ T cells and as an early activated marker)
8. Add 20 µl of anti-biotin microbeads in flow buffer (15 min, 4 °C)
9. Wash cells with cold Flow Buffer and centrifuge (350 g, 6 min, 4 °C)
10. Resuspend the pellet in surface stain antibody cocktail with viability dye (30 min, 4 °C)
11. Wash cells with cold Flow Buffer and centrifuge (350 g, 6 min, 4 °C)
12. Resuspend the pellet in fix/permeabilization solution (20 min, 4 °C)
13. Wash cells with cold Flow Buffer and centrifuge (350 g, 6 min, 4 °C)
14. Resuspend the pellet in intracellular stain antibody cocktail (30 min, 4 °C)
15. Wash cells with cold Flow Buffer and centrifuge (350 g, 6 min, 4 °C)
16. Resuspend the pellet in 500 µl of manual separation buffer

### Magnetic enrichment and acquisition on flow cytometer

17. Cells are enriched, either manually based on protocol modifications suggested by the manufacturer (MS columns with 23 g syringe needles and an OctoMACS magnet) or automated on a MACSQuant Analyzer 10 using running mode “EnrichS”, and acquired on a MACSQuant Analyzer 10 (Miltenyi Biotec, San Diego, CA).

### Data analysis

**Fig. 1:**
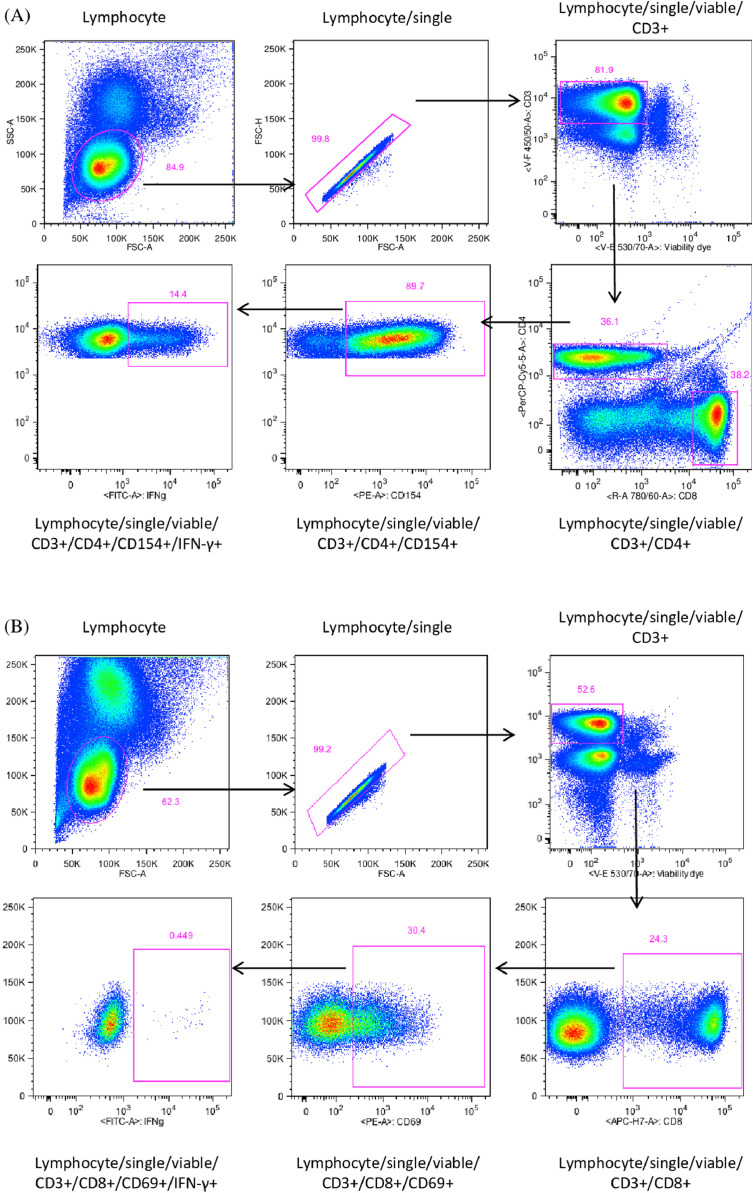
Gating strategy for antigen-stimulated PBMCs. Cells were gated for lymphocytes by Forward and Side scatter (FSC:SSC), and then based on viability and the lineage marker CD3. Enriched antigen-specific cells were gated based on expression of CD4 and CD154 (A) or CD8 and CD69 (B), and then gated for IFN-γ.

18. The quantitative analysis is performed using FlowJo v10 (Treestar, Ashland, OR). The frequency of cells is expressed as the percentage of parent cells (i.e., frequency of CD4^+^IFN-γ^+^ T cells is expressed as percentage of the CD4^+^ T cell population). The gating strategy is shown in Fig. 1. Cells are first gated for lymphocytes by Forward and Side Scatter (FSC:SSC), singlets, and then based on viability and lineage marker CD3. Enriched antigen-specific cells are gated by co-expression of CD154 and CD4 or CD69 and CD8, and subsets evaluated for positivity for IFN-γ (Fig. 1).

## Method validation

Understanding immunity is essential for improving disease intervention strategies or vaccine design. The characterization of lymphocyte subsets participating in immune responses induced by either immunization or infection produces the data that hold the promise to reveal immune mechanisms of protection or pathogenesis. The assessment of cytokines can be done either based on measuring secreted cytokines of bulk stimulated leukocytes (or pre-sorted/enriched cell subsets) by multiplex assays or ELISA, or by measuring the cytokines at the cellular level using gene chip assays, RNAseq, or flow cytometry (cytokine capture assays or intracellular staining). Flow cytometry has been a particularly useful method as it allows the identification and characterization of cell populations involved in an immune response. Characterization of functional cell subsets is accomplished by staining for cell surface markers specific for leukocyte lineages, markers of activation/differentiation, and functional markers such as intracellular detection of cytokines. Unfortunately, the frequency of antigen-specific cells is often at or below the detection threshold of conventional flow cytometry [1]. The technical challenge is to amplify the signal of the antigen-specific cells well above the usual detection threshold. There are several options depending on the source of the samples to reduce the background noise thus allowing rare cell detection: (1) tetramer staining in case where the HLA haplotypes of the donors are known; (2) design flow cytometric panels reserving bright fluorochromes for the identification of the low frequency population(s) and processing/acquiring large sample sizes; (3) design strategies such as extensive cleaning procedures prior to sample acquisition to reduce instrumentation-associated noise and extended flushing of the flow cell between samples to reduce the sample carryover. One approach for amplifying the signal of antigen-specific cells well above the threshold is to use the expression of activation marker as identifiers of antigen-specific cells [2], [3], [4], [5], [6]. Magnetic enrichment of activation marker CD154 positive cells prior to the flow cytometric analysis greatly increases the signal strength because most of the cells analyzed are antigen-specific. This method has largely been used for cells after surface staining [7], and we previously used it to enrich follicular helper T cell in a vaccine study [8]. CD69 is an early-activated marker expressed by CD4^+^ and CD8^+^ T cells. Its rapid expression (<2 h post-activation) on a broad range of immune cells (T cells, B cells, NK cells) makes it amenable for the early detection of T-cell activation and for subset activation analyses [9]. However, the background expression of CD69 can overestimate the frequencies of antigen-specific T cells as TCR-independent activation (bystander) can also trigger expression of CD69 [10,11]. It may therefore be useful to either use a different activation marker such as CD154 or add a second activation marker in the gating strategy to increase the specificity. The use of magnetic enrichment of live cells stained for cell surface markers is possible [1,12], but the feasibility of this approach has not been tested for samples that have been prepared for intracellular staining, *i.e*., fixed and permeabilized prior to magnetic enrichment. The current study sought to determine whether magnetic enrichment for lymphocyte activation marker such as CD154 or CD69 is feasible for cells that have been prepared for intracellular staining. Fixation can increase cell aggregation and non-specific binding of ligands, both of which could interfere with an efficient magnetic bead separation. Our data demonstrate feasibility without impairing the enrichment process using either manual or automated methods, such as enrichment on a flow cytometer with a built-in column, like the MacsQuant™.

### Magnetic enrichment of fixed/permeabilized cells is possible without increasing the non-specific background

**Fig. 2:**
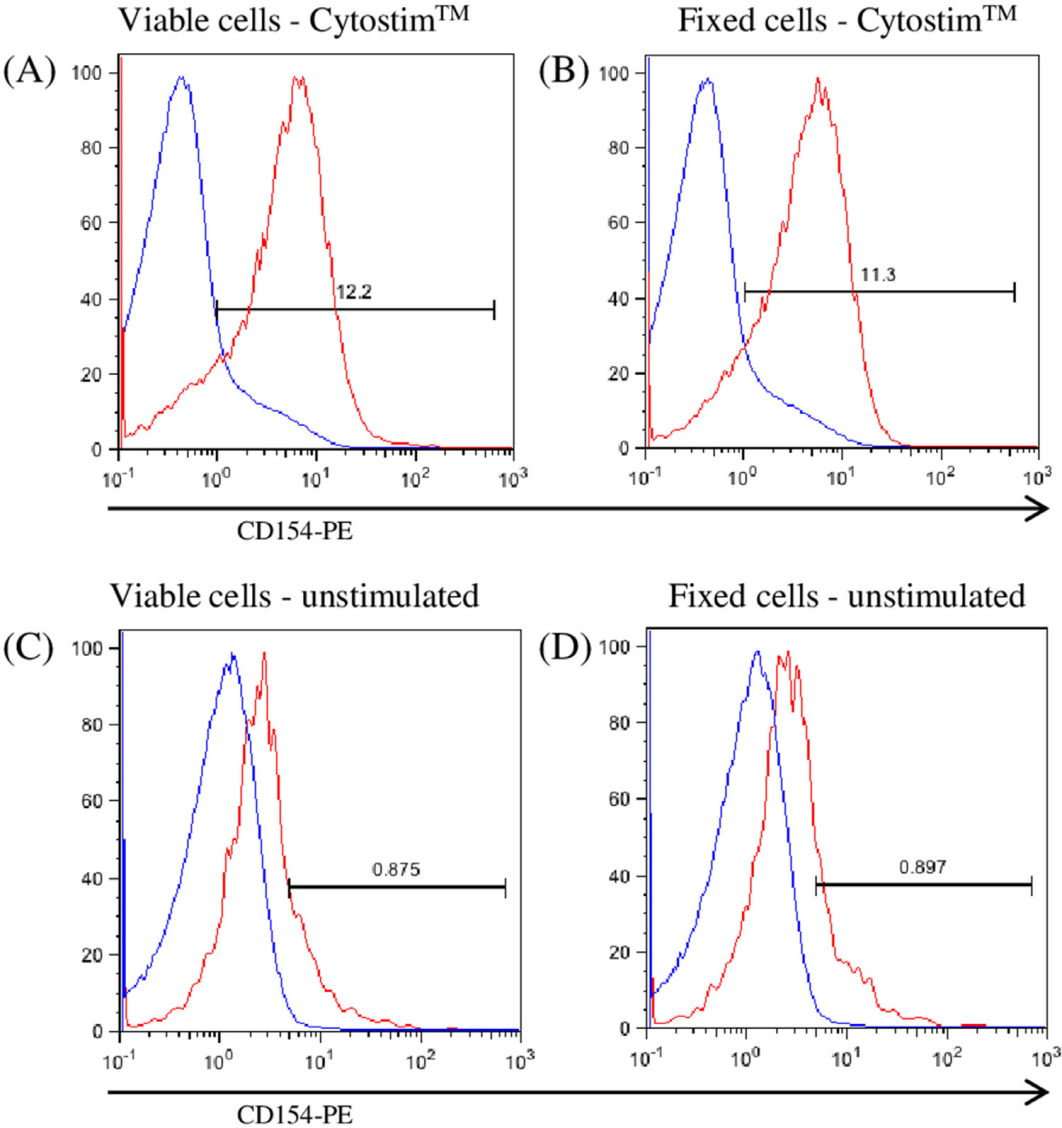
Magnetic enrichment of fixed/permeabilized cells does not result in non-specific enrichment of cells. PBMCs were stimulated with Cytostim™ reagent overnight or left unstimulated and subsequently stained and enriched for CD154 with or without prior fixation. Enrichment from Cytostim™-stimulated viable PBMCs (A) or fixed PBMCs (B) resulted in a significant increase in the frequency of CD154^+^ cells. The histogram overlays show the frequency of CD154^+^ T cells in unenriched (blue line) and enriched (red line) cultures. Specificity of enrichment on fixed samples is demonstrated by CD154 staining on unstimulated cells without (blue line) and with enrichment (red line) of viable (C) or fixed unstimulated cells (D), showing the same background after enrichment. Representative data from two experiments with four donors are shown.

As proof of concept, we first polyclonally stimulated PBMCs with SEB or Cytostim™ and compared the enrichment efficiency and specificity using either viable or fixed/permeabilized cells (Table 1). Staining of SEB-stimulated live cells results in a mean frequency of 10.9 ± 3.2% CD154^+^ cells while staining of fixed cells for CD154 resulted in 15.1 ± 2.7% cells. Enrichment of viable cells based on CD154 resulted in 73.1 ± 5.6% positive cells, and similarly, enrichment of fixed cells yielded 78.5 ± 7.2% CD154^+^ T cells. There was no statistically significant difference between viable or fixed cells after SEB stimulation, or after Cytostim™ stimulation (Table 1). These data indicate that enrichment does not change the specificity and efficiency when using fixed /permeabilized cells. CD154 staining of either unstimulated or Cytostim™-stimulated viable (Fig. 2A, C) or fixed cells (Fig. 2B, D) demonstrates that enrichment of fixed/permeabilized cells based on CD154-expression is possible. The number of antigen-specific cells in the enriched fraction of viable and fixed cells is similar (Fig. 2A, B), as well as the amount of background in unstimulated cells (Fig. 2C, D).

**Table 1:** Frequency of CD4^+^CD154^+^ cells before (UnEnriched) and after (Enriched) CD154 based magnetic enrichment of viable or fixed/permeabilized cells after SEB or Cytostim™ polyclonal stimulation.

Experimental Groups	SEB		Cytostim™	
	Viable cells	Fixed/perm. cells °	Viable cells	Fixed/perm. cells °
UnEnriched	10.9 ± 3.2	15.1 ± 2.7	16.4 ± 3.2	13.2 ± 1.7
Enriched ^⁎⁎⁎^	73.1 ± 5.6	78.5 ± 7.2	77.8 ± 3.7	76.0 ± 4.0

Data are expressed as mean% ± SEM of CD3^+^ CD4^+^ CD154^+^ cells after in vitro stimulation with SEB or Cytostim™ polyclonal antigens. Values from unstimulated cultures have been subtracted. Data from two experiments with four different blood donors.

*** Enrichment resulted in a significantly higher frequency of CD3^+^ CD4^+^ CD154^+^ cells in enriched samples for each group (*p* < 0.001).

° No statistical differences between viable cells and fixed/permeabilized cells in both stimulated groups (Wilcoxon-Mann-Whitney test).

### Enrichment based on activation marker CD154 for CD4^+^ T cells or CD69 for CD8^+^ T cells significantly increases the sensitivity of the flow cytometry analysis and does not skew the composition of functional cell subsets

**Fig. 3:**
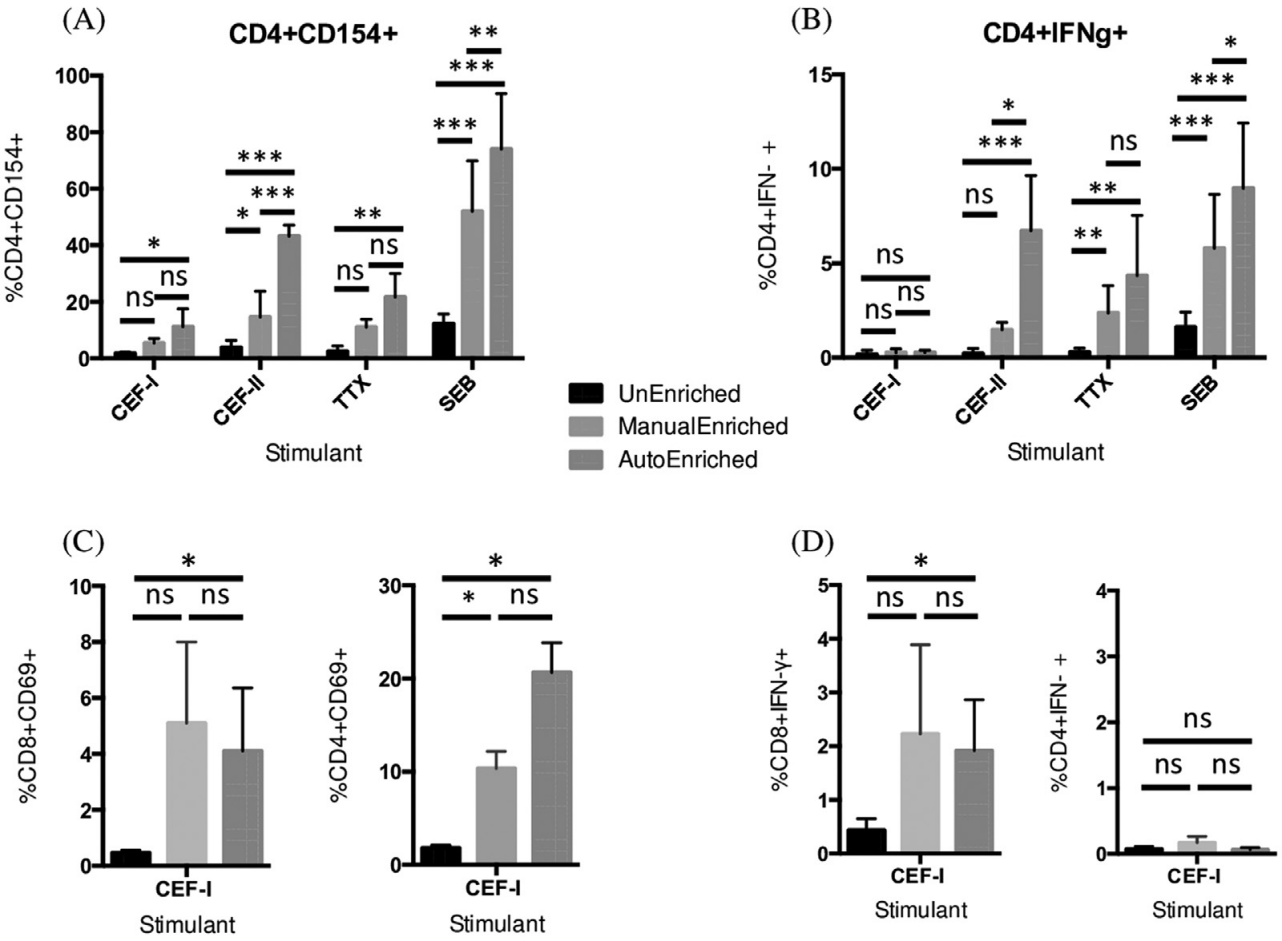
CD154 and CD69-based magnetic enrichment of fixed cells significantly increased the sample size of antigen-specific cells, thus enabling the detection of low-frequency subsets. (A) Frequency of CD4^+^CD154^+^ cells increased significantly after CD154 enrichment (two-way ANOVA, *p* < 0.0001 for both methods of enrichment; multiple T-tests with Sidak-Bonferroni method). (B) CD154 enrichment increased the frequency of IFN-γ producing cells within the CD4^+^ population (two-way ANOVA, *p* < 0.0001 for both methods of enrichment; multiple T-tests with Sidak-Bonferroni method). (C) Frequency of CD8^+^CD69^+^ cells and CD4^+^CD69^+^ cells increased significantly after CD69 automated enrichment (Mann-Whitney test). (D) CD69 enrichment increased the frequency of IFN-γ producing cells within the CD8^+^ population after CEF class-I stimulation (Mann-Whitney test). Data represent mean percentage of positive cells +/- SEM. Values from unstimulated cultures have been subtracted. Data from five experiments with six different blood donors for (A) and (B). Data from three experiments with four different blood donors for (C) and (D). * *p* < 0.05 ** *p* < 0.01 *** *p* < 0.001, ns = statistically not significant.

PBMCs stimulated with various antigens were analyzed by flow cytometry after CD154 enrichment. An aliquot of the samples was analyzed without prior enrichment to determine if enrichment provides a measurable advantage over standard flow cytometry by increasing the frequency of antigen-specific T cells producing IFN-γ to a point where the sample size is statistically significant thus allowing further in-depth characterization. To this end, cells were stimulated with various antigens and stained for flow cytometric analysis. Data were acquired from samples with or without magnetic enrichment (either manually on an OctoMACS™ magnet or automatically using the built-in separation columns of the MacsQuant ™ on the “EnrichS” mode). To ensure that the results were valid for a variety of antigens, we stimulated the cells with CEF class-I, CEF class-II, tetanus toxoid (TTX), and the polyclonal stimulator SEB. Media controls were used as negative controls. We found a significant increase of CD4^+^ CD154^+^ T cells (two-way ANOVA *p* < 0.0001) after CD154^+^ magnetic enrichment (Fig. 3A and Table 2). This observation parallels previous results [8] when using unfixed, non-permeabilized cells showing that enriching viable cells based on CD154 expression results in a significant increase in the frequency of antigen-specific cells. Importantly, our data demonstrate that 1) enrichment based on CD154 is possible using fixed, permeabilized samples and, 2) enrichment increases the sample size of functional, antigen-specific cells within the CD4^+^ population. By measuring the frequency of IFN-γ ^+^ responder cells within the CD4^+^ population (Fig. 3B and Table 2), we observed that CD154 enrichment had a significant impact on the frequency of CD4^+^ IFN-γ ^+^ cells (two-way ANOVA, *p* < 0.0001). As expected -and serving here as specificity control - we saw no increase in CD4^+^ IFN-γ producing cells after CEF class-I stimulation, which only includes CD8^+^ T cell epitopes.

**Table 2:** Frequency of CD4^+^CD154^+^ cells before (UnEnriched) and after manual (ManualEnriched) or automated (AutoEnriched) CD154 based magnetic enrichment of fixed cells.

Experimental Groups	CD4^+^CD154^+^T cells		
	CEF class-II	TTX	SEB
UnEnriched	3.8 ± 2.5	2.4 ± 2.0	12.3 ± 3.4
ManualEnriched	14.6 ± 9.2 *	11.1 ± 2.8 ^ns^	52.0 ± 17.8^⁎⁎⁎^
AutoEnriched	43.3 ± 3.8 ^***/***^	21.7 ± 8.3 ^**/ns^	74 ± 19.6 ^***/**^

Experimental Groups	CD4^+^ IFN-γ^+^ T cells		
	CEF class-II	TTX	SEB
UnEnriched	0.2 ± 0.3	0.3 ± 0.2	1.6 ± 0.8
ManualEnriched	1.5 ± 0.4 ^ns^	2.3 ± 1.5 ^⁎⁎^	5.8 ± 2.9 ^⁎⁎⁎^
AutoEnriched	6.7 ± 2.9 ^***/*^	4.4 ± 3.2 ^**/ns^	9.0 ± 3.5 ^***/*^

Data expressed as mean percentage ± SD of cells after stimulation with CEF class-I, CEF class-II, TTX and SEB. Values from unstimulated cultures have been subtracted. Data from five experiments with six different blood donors.

Multiple T-tests with Sidak-Bonferroni method are represented in the table (* *p* < 0,05 ** *p* < 0,01 *** *p* < 0,001 ns = not significant). ManualEnriched line shows statistical significance with UnEnriched samples. AutoEnriched line shows statistical significance with UnEnriched/ManualEnriched samples.

Since only a small fraction of activated CD8^+^ T cells express CD154 [13], we stimulated PBMCs from four different donors with CEF-I and enriched cells based on CD69 expression. CD69 is an early-activation marker expressed by CD4^+^ and CD8^+^ T cells [9]. We first validated the efficacy of CD69 enrichment by showing significant enrichment of CD3^+^CD8^+^CD69^+^ cells using an automated method, i.e.*,* the MacsQuant (*p* = 0.04, Mann-Whitney test) and CD3^+^CD4^+^CD69^+^ cells with both manual and automated enrichment (*p* = 0.029, Mann-Whitney test) (Fig. 3C). Next, we measured the proportion of IFN-γ producing cells within the CD8^+^ and CD4^+^ populations. There was a significant increase in the frequency of CD8^+^ IFN-γ^+^ cells, when using the automated enrichment method (*p* = 0.02, Mann-Whitney test) (Fig. 3D), and, as expected, no increased in the frequency of CD4^+^ IFN-γ^+^ cells since they were not stimulated by the CEF class-I peptide pool.

Both methods of enrichment were efficient, with a higher statistical significance for the automated enrichment. To address the question of technical variability introduced by the manual method, we used a technical triplicate from one blood donor after CEF class-II stimulation. The coefficient of variation (% CV), calculated by dividing the standard deviation by the mean, resulted in % CV = 1.1 for the unenriched sample, 0.4 for the manually enriched sample, and % CV = 0.6 for the automated enriched sample, suggesting that enrichment did not increase, but actually reduce variability.

**Fig. 4:**
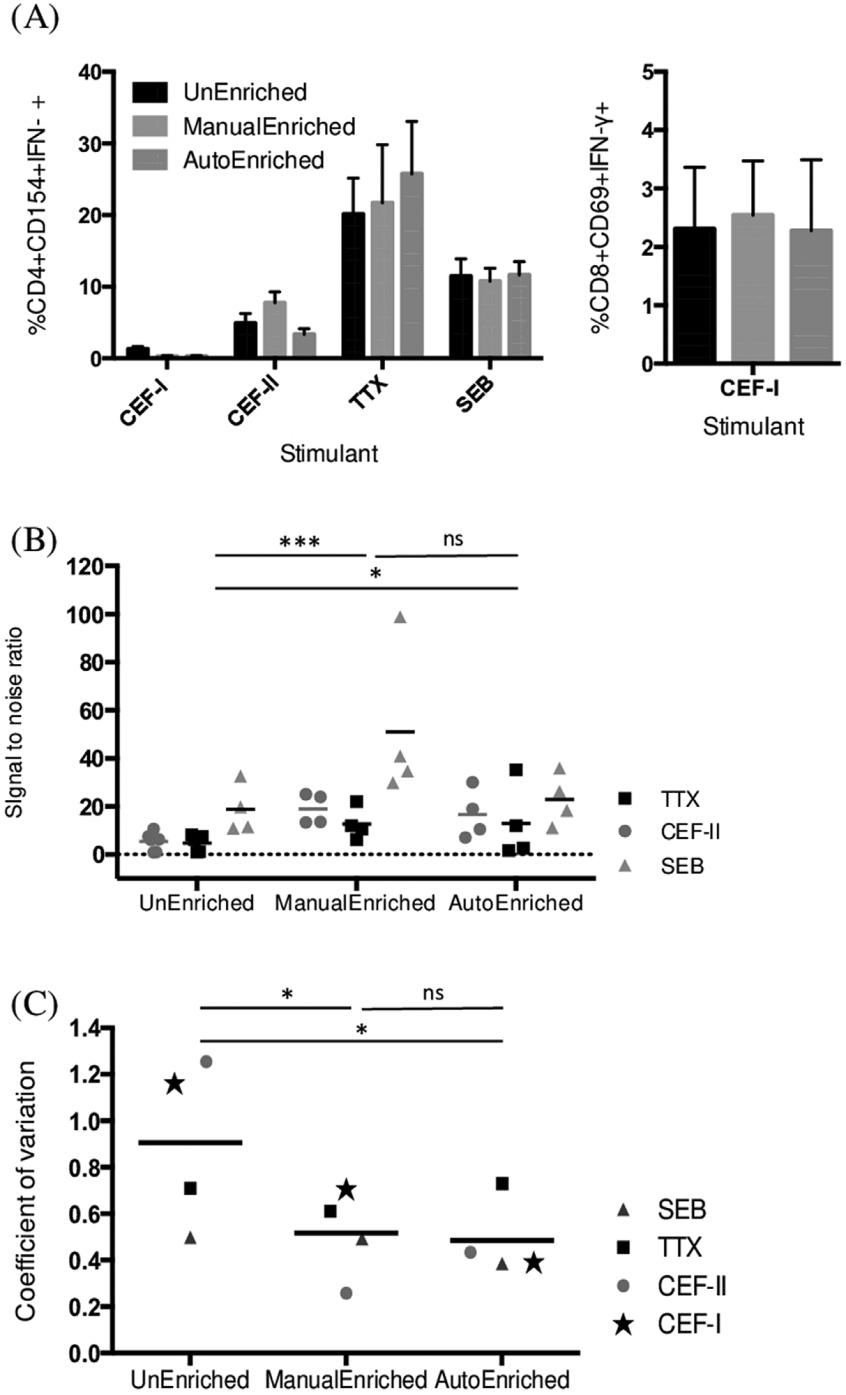
Composition of functional cells, signal-to-noise ratio and coefficient of variation of CD4^+^ IFN-γ^+^ T cells after antigen stimulation. (A) Frequency of IFN-γ^+^ responder cells within the CD4^+^CD154^+^ population or the CD8^+^CD69^+^ population is not modified after enrichment (two-way ANOVA *p* = 0.525). (B) The signal-to-noise ratio for the CD4^+^IFN-γ^+^ T cells population shows a significant increase after enrichment performed either manually (two-way ANOVA, *p* = 0.0004) or automated (two-way ANOVA, *p* = 0.0328). (C) Overall decrease of the coefficient of variation after enrichment performed either manually (T-test, *p* = 0.036) or automated (T-test, *p* = 0.019). Data represented mean +/- SEM. Data from five experiments with six different blood donors.

Another important consideration is whether enrichment skews the composition of cell subsets and skews the ratio of the responder population. If enrichment were to introduce a bias, this approach could only be cautiously used for analyzing T cells subsets. For this purpose, we determined within the CD4^+^ CD154^+^ population the relative frequencies of IFN-γ producing cells (Fig. 4A left) and found no difference before and after magnetic enrichment (*p* = 0.525, two-way ANOVA). Similarly, we observed no difference in the relative frequencies of CD8^+^CD69^+^ IFN-γ^+^ cells before or after CD69 enrichment (*p* = 0.657, Mann-Whitney test) (Fig. 4A right).

Finally, to determine whether enrichment introduces additional noise or variability, we calculated the signal-to-noise ratio and the coefficient of variation for the CD4^+^IFN-γ^+^ T cells population after different stimulations. The signal-to-noise ratio, calculated by dividing the cell frequency of CD4^+^IFN-γ^+^ T cells under the antigen-stimulated condition with the same readout taken from the unstimulated condition, was significantly increased after manual and automated enrichment (two-way ANOVA, *p* = 0.0004 and *p* = 0.0328 respectively, Fig. 4B). The ratio for TTX, which is a single-antigen stimulation, increased from 4.81 ± 1.55 (mean ± SEM) in unenriched samples to 12.69 ± 3.34 in manually enriched samples and to 12.87 ± 7.82 in automated enriched samples. The coefficient of variation was significantly decreased after manual and automated enrichment (T-test, *p* = 0.036 and *p* = 0.019 respectively, Fig. 4C), confirming that enrichment helped to reduce the variability between biological samples.

In conclusion, manual and automated enrichment significantly increase the sample size of functional antigen-specific cells without skewing the composition of functional cells and with an overall higher efficiency in the automated method. By increasing the signal-to-noise ratio and reducing the variability, both methods of enrichment provide a helpful approach for the discrimination of antigen-specific cells.

### Additional information: power analysis

**Fig 5:**
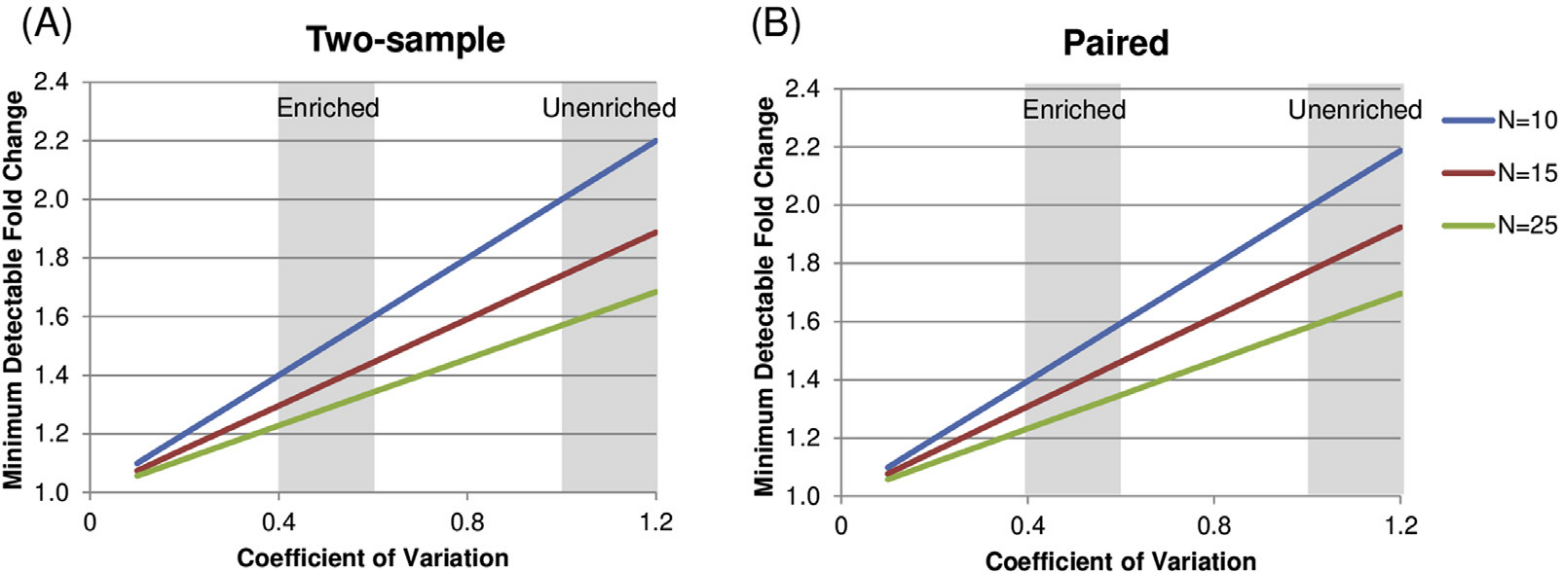
Power analysis comparing effect size and CV. (A) The minimum difference in means between a reference group and a test group, expressed as a fold change, is shown as a function of the variability, expressed as the CV, for study sample sizes of 10, 15, and 25 subjects per group, using a two-sample comparison (B) the minimum difference in means, expressed as a fold change, as a function of CV for sample sizes of 10, 15, and 25 subjects per group, using a paired comparison. CV value ranges corresponding to unenriched and enriched conditions are highlighted.

Our findings demonstrate that enrichment can increase the signal-to-noise ratio and reduce the variability in the measured frequencies of rare cell types in both technical replicates and in biological samples. To assess the practical value of this reduction in variability, we carried out a power analysis to determine how the minimum effect size that is reliably detectable for a given study sample size is altered by a reduction in the CV, based on a power of 0.80 and a significance value of 0.05, for a range of sample sizes. The power analysis assumes all samples have comparable variability following enrichment to demonstrate how a reduction in variability improves the sensitivity of the assay to detect differences in immune responses. Users are advised to obtain a reliable estimate of the variability in their own assay for a range of cell frequencies relevant to their sample set before using this power analysis to determine what effect size might be reliably distinguished through their assay. The minimum effect size was expressed as the difference in means between a reference group (*μ*_1_) and a test group (*μ*_2_), calculated as a fold change ($A=\;\frac{\mu_{2}-\mu_{1}}{\mu_{1}}$). The relationship between the minimum detectable fold change, CV, and effect size can be expressed as *A* = (CV x effect size) + 1. Effect size here is expressed as a difference in means between a reference group and a test group, calculated as a fold-change difference. We carried out this analysis for both two-sample comparisons (Fig. 5A), which compare measurements between two groups, and paired comparisons (Fig. 5B), which compare two measurements from the same group, such as from samples collected at different time points. Our power analysis shows that in the case of unenriched samples, which show a CV ranging from 1.0 to 1.2, the minimum detectable difference ranges from 1.6 fold to 2.2 fold, corresponding to a 60% to 120% increase in the measurement from one group to another, depending on the sample size. By contrast, enrichment results in CV values around 0.40 to 0.60, which corresponds to a minimum detectable difference ranging from 1.25-fold and 1.6-fold, corresponding to a 25% to 60% difference in means depending on the sample size. We found that the results were similar between the two-sample and paired comparisons. This analysis confirms that enrichment can substantially improve the resolution of detecting differences between study groups or across time points for rare or low-frequency cell types.

## Declarations

### Authors’ contributions

MM performed experiments and compiled the manuscript with EB-L. FF and EHD performed experiments, SC performed the statistical analysis, KB and EB-L designed the experiments. MM, FF, EHD, SC, KB reviewed and edited the manuscript.

### Availability of data and materials

The data and detailed protocol can be made available by the corresponding author upon request.

### Disclaimer

Material has been reviewed by the Walter Reed Army Institute of Research. There is no objection to its presentation and/or publication. The opinions or assertions contained herein are the private views of the authors, and are not to be construed as official, or as reflecting the views of the Department of the Army or the Department of Defense. This paper has been approved for public release with unlimited distribution. The investigators have adhered to the policies for protection of human subjects as prescribed in AR 70–25.

## Funding

This work was supported by the Military Infectious Disease Research Program.

## Declaration of Competing Interest

The authors declare that they have no conflict of interest.
